# Typical median effective radiation doses using an anthropomorphic bone fracture phantom for initial radiographic skeletal surveys in the investigation of suspected physical abuse

**DOI:** 10.1007/s00247-022-05456-x

**Published:** 2022-08-22

**Authors:** Edel Doyle, Matthew R. Dimmock, Kam L. Lee, Peter Thomas, Richard B. Bassed

**Affiliations:** 1 Department of Forensic Medicine, Monash University, 65 Kavanagh Street, Melbourne, Southbank, VIC 3006 Australia; 2 Medical Imaging and Radiation Sciences, Monash University, Melbourne, Australia; 3 School of Allied Health Professions, Keele University, Staffordshire, UK; 4 Australian Radiation Protection and Nuclear Safety Agency, Yallambie, VIC Australia; 5 Academic Programs, Victorian Institute of Forensic Medicine, Melbourne, VIC Australia

**Keywords:** Child, Diagnostic reference level, Infant, Inflicted injury, Nonaccidental injury, Physical abuse, Radiation dose, Radiography, Skeletal survey

## Abstract

**Background:**

A series of 31 radiographs is recommended by the Royal College of Radiologists (RCR) when investigating suspected physical abuse (SPA).

**Objective:**

To determine the radiation dose delivered for skeletal surveys performed for SPA in Victorian radiology departments based on their local protocols.

**Materials and methods:**

A 5-year-old paediatric bone fracture phantom was radiographed at five radiology sites using both the RCR recommended protocol and, where applicable, the local departmental SPA protocol. The radiation doses were measured and recorded. These were scaled down to estimate the effective radiation doses for a 2-year-old child at each site and the associated radiation risks estimated.

**Results:**

The median effective dose for all radiographic projections in the RCR skeletal survey radiographic series was 0.09 mSv. The estimated risk of radiation-induced cancer and radiation-induced death from cancer for 2-year-old children is classified as “very low,” with girls having a higher risk than boys.

**Conclusion:**

The median effective radiation dose for the RCR skeletal survey for imaging in SPA was 0.09 mSv resulting in a “very low” additional risk of radiation-induced cancer. The authors will now aim to ascertain whether whole-body CT skeletal survey can replace the radiographic series for imaging in SPA while maintaining a comparable radiation dose.

**Supplementary Information:**

The online version contains supplementary material available at 10.1007/s00247-022-05456-x.

## Introduction

Nonaccidental injury (NAI) was first described by Dr. John Caffey in 1946 [1] and is more recently referred to as suspected physical abuse (SPA) or inflicted injury [2]. In recent decades, professional bodies have published guidelines on recommended radiographic projections to best demonstrate the highly specific injury patterns associated with SPA; this is collectively referred to as a radiographic skeletal survey. The Royal College of Radiologists (RCR) standard recommends a series of 31 radiographic projections [2], as well as follow-up imaging. SPA skeletal surveys are forensic in nature and are performed for medicolegal, rather than solely medical, purposes. In 2008, the RCR and Royal College of Paediatrics and Child Health suggested that this radiographic series would typically have an effective radiation dose of 0.9–1.8 mSv [2]. The RCR also advocates follow-up imaging to better demonstrate healing fractures and some departments continue to perform scintigraphy, therefore the effective dose can be as high as 3 mSv [3]. Due to the frequency of uncooperative younger patients, the skill level of the radiographer in achieving diagnostic images at the lowest patient radiation dose is of particular importance [4]. A wide variability in the quality of radiographs, with skull radiographs often being the poorest of these, has also been reported [5]. Although best practice would suggest that all radiographic images acquired, including repeated images, are included for reporting in forensic cases, this may not be common practice, yielding a higher cumulative radiation dose than would be calculated using the images sent to the picture archiving and communication system (PACS).

Children have an increased risk of developing cancer when exposed to radiation as their organs are more radiosensitive and they have a longer lifespan during which to develop cancer. In Australia, the estimated risk of being diagnosed with a blood cancer is 1 in 1,904 in children younger than 5 years old [6]. This is estimated to be half the risk of developing any type of cancer, so the risk is 1 in 1,000 for children younger than 5 years old and 1 in 400 for children younger than 15 years old. An additional lifetime risk of developing cancer of approximately 0.01–0.03% is associated with a radiographic skeletal survey [7]. The benefits of diagnosing SPA, which may be fatal, outweigh the risks associated with ionising radiation [7–9], as SPA is determined to be the most prevalent cause of childhood deaths with modifiable causes in England [7]. Nevertheless, careful consideration should be taken to minimise the dose delivered.

The World Health Organization (WHO) has estimated that nearly 3 in 4 children ages 2–4 years regularly suffer physical punishment and/or psychological violence at the hands of parents and caregivers and that some of the estimated 40,000 homicide deaths in children younger than 18 years old each year are likely due to child maltreatment [10]. During 2019–2020, 174,700 (1 in 32) Australian children received child protection services (investigation, care and protection order and/or were in out-of-home care) with Aboriginal or Torres Strait Islander children 8 times more likely to have received these services [11]. In 2019–2020, physical abuse was the primary type of abuse substantiated for 14% of children in Australia [11]. From 2015 to 2020, the rate of substantiated physical abuse in children remained constant at 1.5 in 1,000 (1 in 650) children [10]. Australian research using hospital morbidity data has shown that 32% of children admitted to hospital in Queensland between January 2003 and December 2006 with an unintentional injury were known to the child protection authorities [12], suggesting that a number of admissions likely to be associated with SPA are not recognised as such [13]. In a recent study in South Australia, it was shown that children known to child protection services had higher mortality rates, but only 2 of the 1,635 deaths listed child maltreatment as a contributing cause [14]. Research has consistently found that the youngest children are the most vulnerable to abuse- and neglect-related deaths [13]. It has been suggested that failing to suspect physical abuse may result in 35% of children presenting to the emergency department due to re-injury and up to 5% may die from subsequent injuries [12]. In 2016, it was reported that 1 in 20,000 children younger than 1 year old in Victoria died from inflicted injuries compared to 1 in 165,000 of children aged 1 to 4 years old [15].

The skeletal survey is considered the imaging gold standard to contribute to an investigation of SPA as “hidden” injuries may be revealed [7]. The skeletal survey routinely includes anteroposterior (AP) and lateral projections of the skull; AP and oblique projections of the chest, including visualization of the ribs; an AP projection of the abdominopelvic cavity, lateral projections of the spine and AP projections of the arms and legs [2, 16, 17]. Supplementary coned projections of the joints including lateral projections of the wrist, elbow, knee and ankle, as well as AP mortise of the ankles and dorsipalmar/dorsiplantar (DP) projections of the hands and feet are routinely taken to assist radiologists in the diagnosis of metaphyseal fractures [2, 16, 17]. Variability in the physical radiographic equipment, exposure factor optimization and the number of images recommended can result in significant differences in radiation dose between clinical centres within a given jurisdiction [8]. Indeed, the International Commission on Radiological Protection (ICRP) recognises that the updating of paediatric diagnostic reference level (DRL) values has been slow in comparison with the rapid development of imaging technology and that there is a need to establish paediatric DRLs [18]. In addition to the uncertainty in the dose associated with planar radiography for investigating SPA, the potential use of low-dose computed tomography (CT) has also been proposed [19]. If CT is to potentially replace planar radiography in establishing SPA, then the optimal effective dose for the scan should be compared to that of the current gold standard, although it is acknowledged that the professional community may accept a higher radiation dose from CT if it results in a higher diagnostic accuracy in identifying fractures suggestive of SPA.

The aim of this study is to propose a radiation dose that is representative of that delivered for skeletal surveys in Victorian radiology departments. To establish the variability in protocols and their associated doses, a phantom study was performed at five local radiology sites. The series of projections recommended by the RCR was taken at each site for use as a reference standard [2], as well as those for sites that had their own local protocol. The results and implications are presented and discussed herein.

## Materials and methods

### Radiographic skeletal survey for SPA

**Fig. 1:**
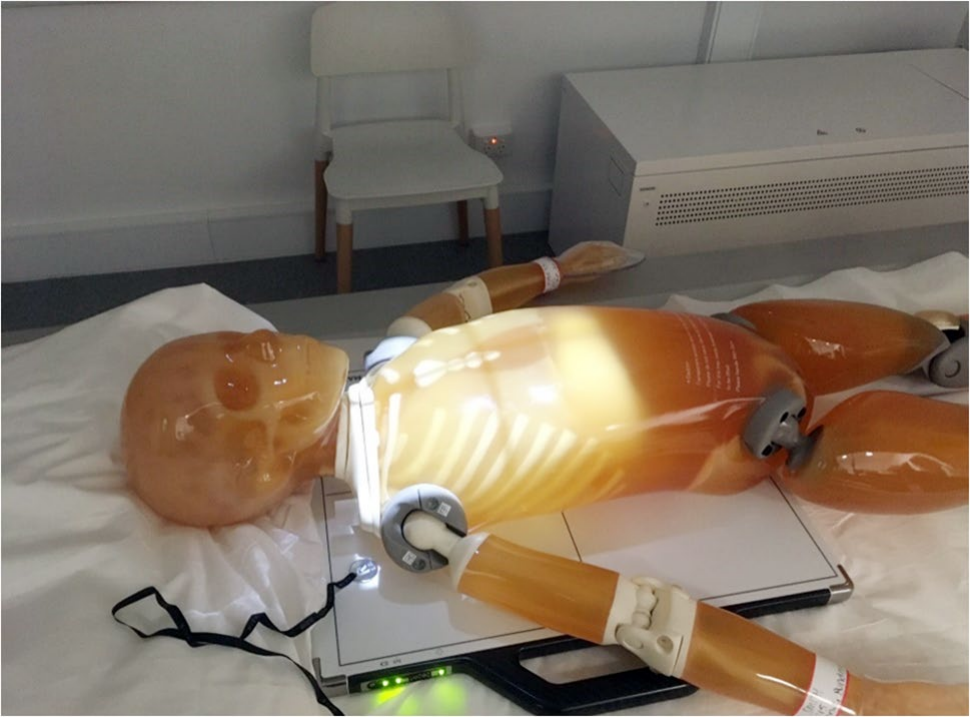
A photograph shows an example of a 5-year-old anthropomorphic bone fracture phantom [20] positioned for an anteroposterior chest radiograph

Permission was sought from five radiology sites to take part in this phantom study. Three of these departments routinely image children, of which two were in dedicated paediatric hospitals. For each centre from which data was collected, radiographic projections for a skeletal survey were acquired in accordance with their departmental SPA policy. Locally recommended exposure factors for a 2-year-old child were used, as this is the most common age in whom SPA imaging is performed. A paediatric anthropomorphic bone fracture phantom with fractures that are highly specific for SPA was imaged at each site. Figure 1 shows the phantom, which simulates a 5-year-old child weighing 19 kg and measuring 110 cm, equivalent to the 5-year-old ICRP reference phantom [20]. Whilst the bone fracture phantom is not representative in terms of the age of patients who most frequently present for skeletal surveys, it is the only commercially available SPA phantom [20, 21] so to address this, the measurements of the irradiated areas for each projection were scaled down to a 2 year old.

### Data collection

At each site, exposure factors including peak tube kilovoltage (kV_p_), tube current–time product, source to image distance (SID) and air kerma area product (KAP) measurements were recorded for each projection. Additional projections were also acquired so that the radiation dose could be compared to those projections recommended by the RCR [2]. The KAP measurements represented the output emitted by the X-ray tube before entering the patient and are a standardized metric to determine radiation dose in general radiography when establishing DRLs [22].

### Dose standardisation

Inter-site KAP measurement variations can arise due to the variation in KAP meter accuracy performance. This is evident from the International Electrotechnical Commission (IEC) specified tolerance (± 35%) [23]. To minimise the variability, the KAP was measured at each site using a PTW Diamentor (PTW Freiburg GmbH, Freiberg, Germany) that was calibrated to a national standard; this enabled the true KAP to be determined for comparison of radiation doses between sites.

**Fig. 2:**
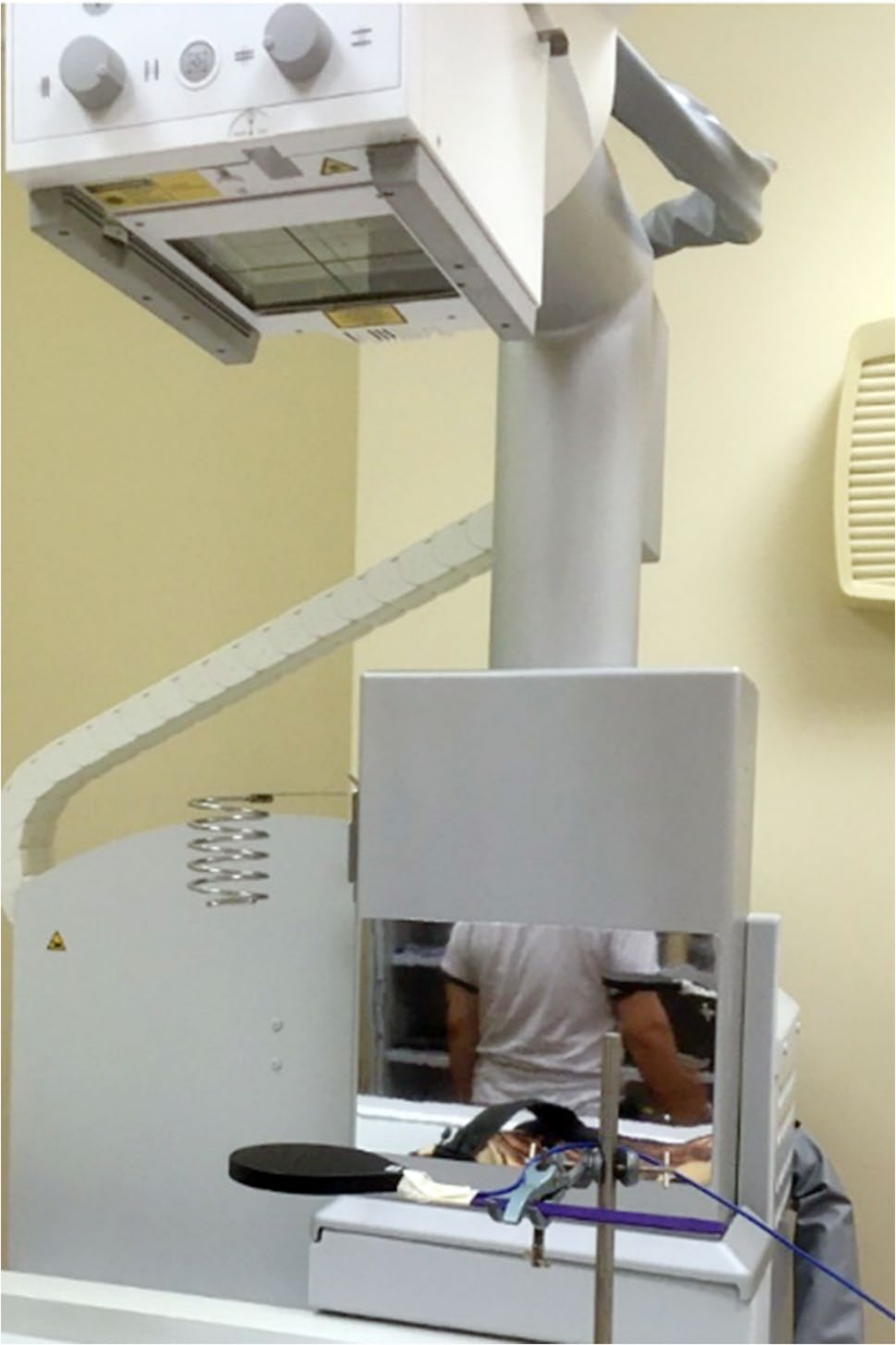
A photograph shows  a Siemens dRF radiographic unit and the 75-cm^3^ pancake chamber

A Siemens dRF radiographic unit (Erlangen, Germany) was used to compare the dose measurements recorded by the PTW Diamentor CD-R KAP meter and a 75-cm^3^ pancake chamber connected to a Nomex electrometer (PTW Freiburg GmbH), which is traceable to the national primary dosimetry standard (Fig. 2).

Using the measurements from the dRF compared to the PTW Diamentor and the dRF compared to the pancake chamber, a calibration factor was determined. This calibration method is called the Substitution Method [24].

The calibrated mobile KAP meter (PTW Diamentor) was brought to each of the five sites and used to acquire readings with a standardised irradiated field of 15.4 cm × 15.6 cm using a series of exposure factors as available on each vendor’s radiographic control panel (Online Supplementary Material 1). These were all manual exposures, as the Automatic Exposure Control is not used when irradiating small children.

This established the calibration factor for each site-specific KAP meter. Each exposure was repeated five times and the mean calculated. The exposure factors included the range used at the clinical sites for the projections performed as part of the skeletal survey series. The calibration factor was applied to the KAP readings for the SPA skeletal survey projections from each site to standardize them all. This allowed comparison of radiation doses between sites by eliminating any error associated with the KAP meters.

### Phantom size correction

Since the irradiated areas were collimated to the relevant anatomical regions, the KAP readings obtained from exposing the 5-year-old phantom would be greater than that of a 2 year old child. As there is no 2 year old anthropomorphic bone phantom available, this was addressed by dividing the KAP measurements by the irradiated area for the 5-year-old phantom to give the air kerma value. Interpolating the surface area data from Table 2.1 in ICRP Publication 143 and applying the total surface area percentage from Table 4.4 in ICRP Publication 89 [21, 25], the correction factors for the different anatomical regions were calculated and are shown in Table 1. In this way, the height and width of the radiographs images were scaled down from a 5 year old to a 2 year old. These scaled-down irradiated areas were then multiplied by the air kerma to give an estimated KAP value for each projection for a 2 year old child. These KAP values were summed to provide an estimated cumulative KAP for the skeletal survey series of radiographs.

**Table 1:** Phantom size correction factors

Region	Correction factor
Head	0.826
Trunk	0.724
Upper extremity	0.672
Lower extremity	0.678

### Radiographic equipment and dose calculation software

The radiographic equipment used to acquire the SPA skeletal survey of the paediatric phantom at each site according to their local protocol is listed in Table 2.

**Table 2:** Specifications of the radiographic equipment used at each of the radiology sites

Site	X-ray tube	Filtration	Detector
1	Siemens Multix Fusion Max (Erlangen, Germany)	Total filtration ≥ 2.5 mm Al/70 kV 0.6 mm Cu	MAX wi-D
2	Siemens Multitom Rax (Erlangen, Germany)	Total filtration ≥ 2.5 mm Al	MAX wi-D
3	GE Optima XR656 (USA) with Siemens X-ray tube (Erlangen, Germany)	inherent filtration of not less than 2.7 mm Al at 70 kV_p_	Flash-PadFlat-Panel Wireless Digital Detector
4	Shimadzu / CMP 200 [GE] X-ray tube (Kyoto, Japan)	2 mm Al at 75 kV	Canon digital detector
5	GE Discovery XR656 (USA) with Siemens X-ray tube (Erlangen, Germany)	2 mm Al at 70 kV	GE wireless detector
Reference	Siemens Luminos dRF (Erlangen, Germany)	2.5 mm Al at 80 kV	Pixium FE 4343F

*Al* aluminium, *Cu*, copper, *kV* kilovolts, *kVp* peak kilovoltage, *mm* millimetre

**Fig. 3:**
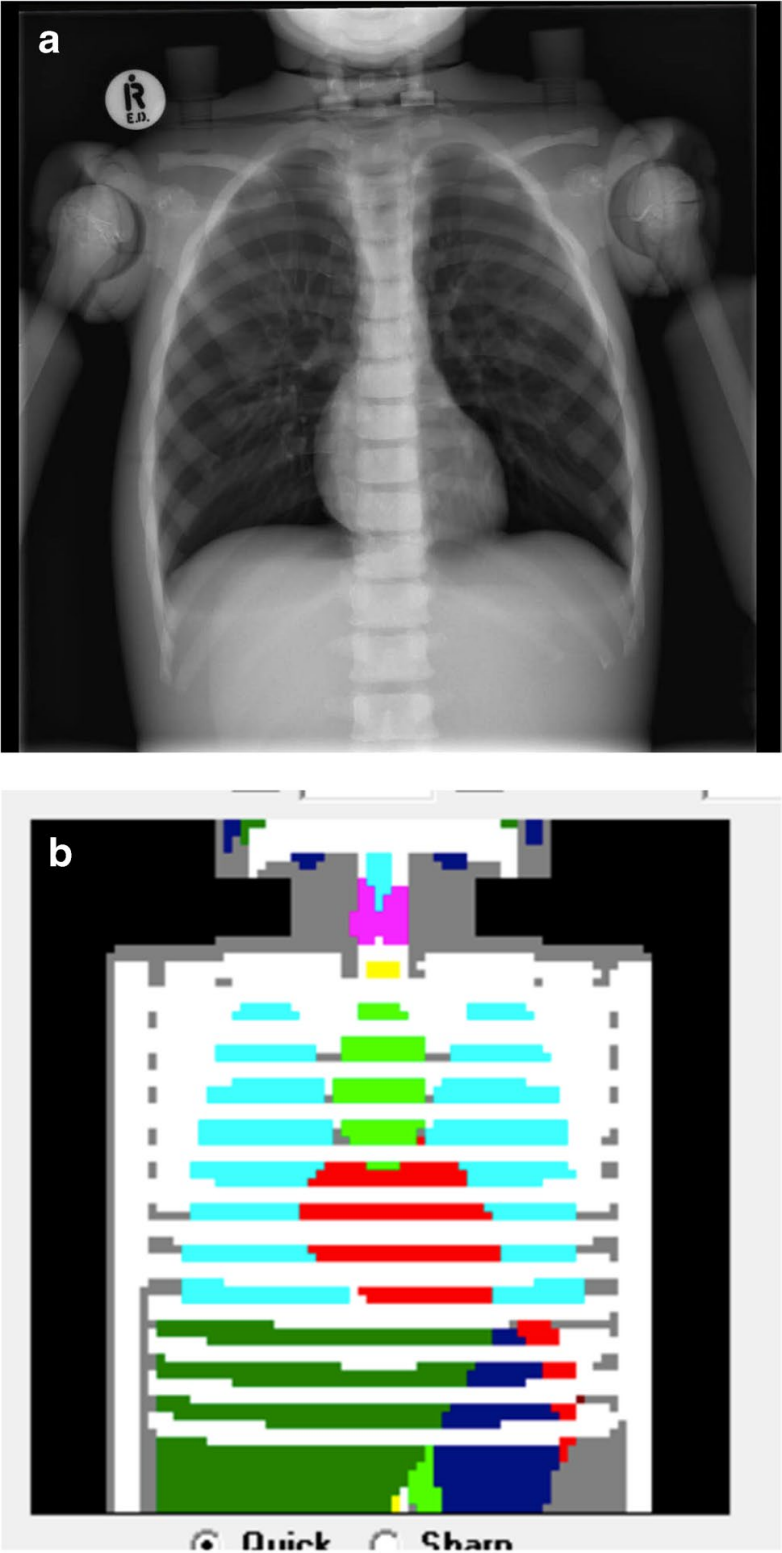
(**a**) An antero-posterior (AP) chest radiograph of the phantom obtained at Site 1 shows the limits of collimation around the irradiated field. **b** The examination entry interface for an AP chest radiograph using the PCXMC software. The different colours represent each of the radiosensitive organs within the irradiated field in (**a**)

The PCXMC (STUK, Vantaa, Finland) software used to calculate the effective dose from each skeletal survey data set is routinely available to medical physicists when calculating organ doses and effective doses to compare patient protocols. The doses can be calculated for 29 organs and tissues and the software can estimate the effective dose with the current tissue-weighting factors of ICRP Publication 103 [26]. The SID and scaled height and width of each radiograph were entered into PCXMC, along with the kV_p_ used to calculate the effective dose (mSv) for each individual projection at each of the five sites. The software calculations are based on the Monte Carlo method to estimate effective radiation doses for each projection, using the height and weight of a 2 year old child, 84 cm and 12 kg [27]. An example of examination entry interface for an anteroposterior chest radiograph is shown in Fig. 3. The effective doses for each projection were then summed to provide an estimated total effective radiation dose (mSv) for the skeletal survey series of radiographs [28].

The local radiographic protocol for SPA projections was used in the three children’s radiology departments as well as locally recommended exposure factors for a 2-year-old child. As these are standard clinical exposure factors, it is accepted that they have been optimized locally to produce such diagnostic quality images that the radiologist is confident to issue a report that will contribute to the medicolegal investigation of suspected SPA. The KAP readings were those produced locally, which would be the values utilized in a calculation of radiation dose by that department. It is appreciated that radiation doses may be optimized more effectively in specialist paediatric radiology departments where SPA imaging should be undertaken with the expertise of the child protection team readily available.

The risk of radiation-induced cancer for 2-year-old children for the RCR skeletal survey protocol at each of the five sites was estimated using Table 12D-1 while the risk of radiation-induced death was estimated using Table 12D-2 published in the Biologic Effects of Ionizing Radiation (BEIR) VII report [29].

## Results

### Calibration of KAP meters

**Fig. 4:**
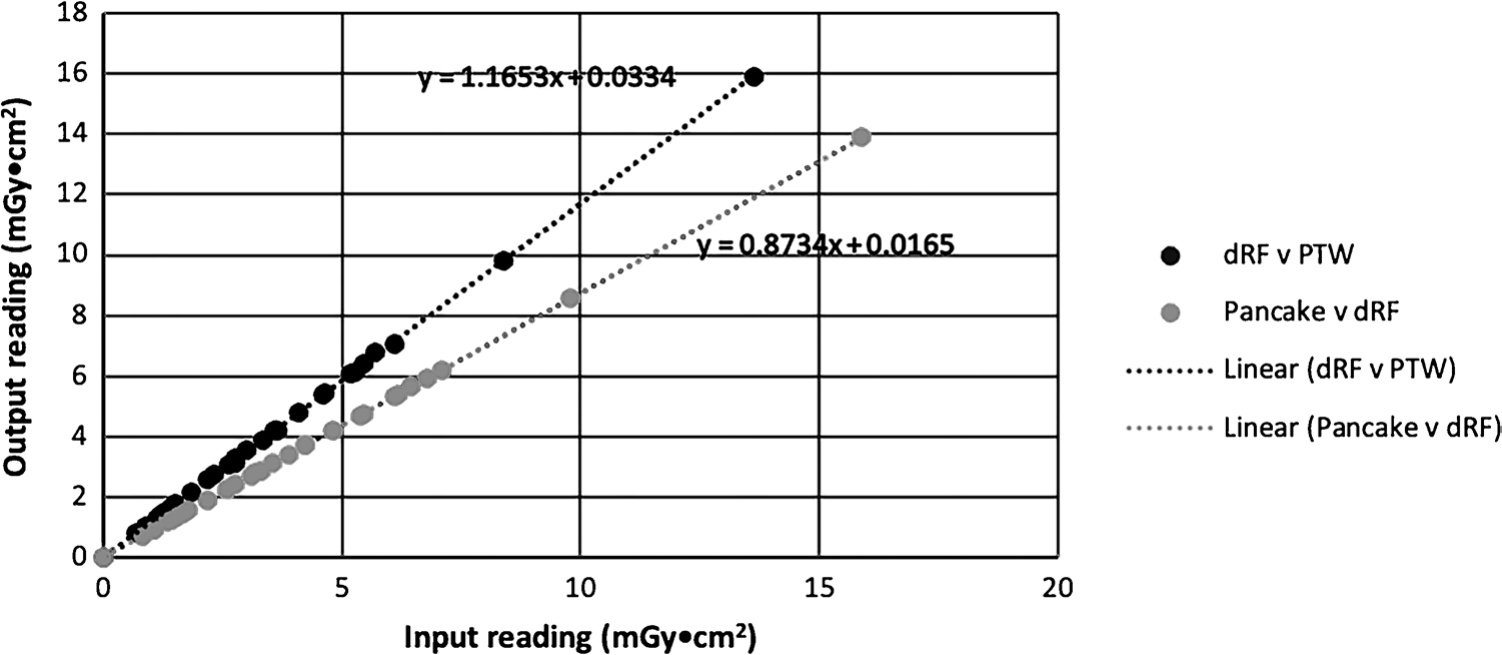
A graph showing linear regression with the lines of best fit between the kerma area product (KAP) measurements using PTW Diamentor and dRF KAP and dRF KAP and pancake chamber

The PTW Diamentor KAP meter was calibrated against the Siemens dRF machine, and the Siemens dRF machine was calibrated against a known secondary standard, namely the PTW pancake chamber. A linear regression was performed to determine the equation of the line of best fit with each of the Siemens dRF machine and pancake chamber measurements (Fig. 4). The substitution method [24] was used to determine the true KAP given by the following equation:

1 $${KAP}_{true}={m}_{2}{m}_{1}{KAP}_{PTW}+{{m}_{2}c}_{1}+{c}_{2}$$

where KAP_true_ is the air kerma area product given by the pancake chamber and field size, *m*_1_ is the slope of the line KAP_dRF_ versus KAP_PTW_, c_1_ is the y-intercept of the line KAP_dRF_ versus KAP_PTW_, m_2_ is the slope of the line KAP_true_ versus KAP_dRF_, and c_2_ is the y-intercept of the line KAP_true_ versus KAP_dRF_

### Calculation of true KAP measurements

**Fig. 5:**
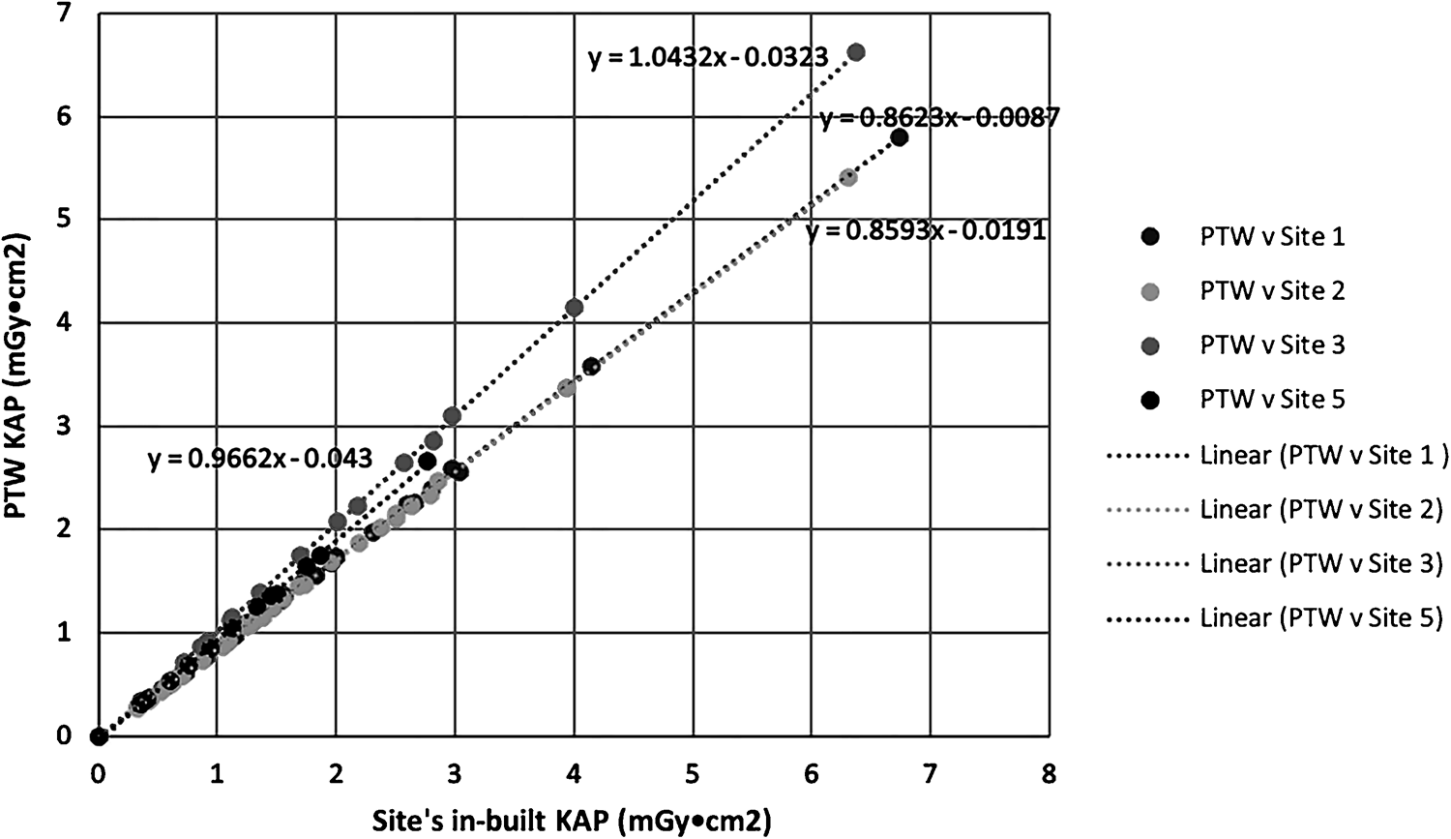
A chart showing linear regression with the lines of best fit comparing the local site’s in-built kerma area product (KAP) measurements against the calibrated PTW Diamentor for sites 1, 2, 3 and 5

It is noted that the attenuation of the built-in KAP meters is negligible. The total filtration of the built-in KAP in the dRF Luminos is 0.5 mm Al eq., which gives a transmission factor of 0.95 at 50 keV. The KAP measurements from each site were converted to the true KAP using the following equation:

2 $${KAP}_{true}={m}_{3}{m}_{2}{m}_{1}{KAP}_{site}+{m}_{1}{{m}_{2}c}_{3}+{{m}_{2}c}_{1}+{c}_{2},$$

where m_3_ is the slope of the line KAP_PTW_ versus KAP_site_, c_2_ is the y-intercept of the line KAP_PTW_ versus KAP_site_. This was performed for each of the exposures acquired for each skeletal survey set of radiographs on the phantom. The mobile KAP meter (PTW Diamentor) was taken to each radiology site to obtain a series of measurements to assess the accuracy of the in-built KAP meter in the X-ray tubes assembly. Graphs of the mobile KAP meter versus local KAP meter were plotted (Fig. 5). Since site 4 does not have a system KAP meter, the conversion equation is Eq. (1).

### SPA protocols

The RCR recommends 31 projections as part of the routine skeletal survey for the investigation of SPA. These were the only projections performed at sites 1 and 4, as they did not have a local departmental protocol to follow. The departmental protocols for sites 2, 3 and 5 were acquired and are presented in Table 3, allowing easy comparison to the RCR-recommended projections. Sites 2, 3 and 5 perform fewer projections than those recommended by the RCR, but their local protocols differ from each other, showing a lack of consistency between departments, as there are no best practice guidelines or recommended projections in Australia, unlike New Zealand [16].

**Table 3:** Projections performed routinely as part of SPA protocol at each of the radiology sites where “✓” means performed

Body part	Projection	Side	(RCR) Site 1	Site 2	Site 3	(RCR) Site 4	Site 5
Skull	AP		✓				
Skull	Lat	L	✓				
Chest	AP		✓				
Chest	Obl.Ribs	R	✓	✓	✓	✓	
Chest	Obl.Ribs	L	✓	✓	✓	✓	
Chest	Lat	L		✓			
Sternum	Lat	L			✓		✓
Abdomen/pelvis	AP		✓				
Spine	Lat L-spine	L		✓			
Spine	Lat T- & L-Spine	L	✓		✓	✓	✓
Humerus	AP	R & L	✓				
Forearm	AP	R & L	✓				
Hand	DP	R & L	✓				
Coned elbow	Lat	R & L	✓			✓	✓
Coned wrist	Lat	R & L	✓			✓	
Femur	AP	Both			✓		✓
Femur	AP	R	✓	✓		✓	
Tib/fib	AP	Both			✓		✓
Tib/fib	AP	R & L	✓	✓		✓	
Foot	DP	R & L	✓				
Coned knee	AP	R & L	✓	✓		✓	
Coned ankle	AP	R & L	✓	✓		✓	
Coned knee	Lat	R & L	✓			✓	
Coned ankle	Lat	R & L	✓			✓	
Total number of projections performed for local SPA skeletal survey protocol			31	24	18	31	17

Where *Tib/Fib* tibia & fibula, *AP* antero-posterior, *PA* postero-anterior, *Lat* lateral, *R* right and *L* left with R&L performed as separate exposures

The variety of exposure factors used for each projection at each site, along with the KAP, have been recorded in Table 4 for comparative purposes.

**Table 4:** Exposure factors (kV_p_ and mAs) selected for a 2-year-old and true kerma area product (KAP) recorded for each projection performed at each site using a 5-year-old anthropomorphic bone fracture phantom. The automatic exposure control was not used for any exposure

Body part	Projection	Side	Site 1	Site 2	Site 3	Site 4	Site 5
		SID (cm)	115	115	100	115	120
			kV_p_/mAs/True KAP (mGy·cm^2^)				
Skull	AP		70/2/47.7	61.5/2.5/27.7	70/8/104.5	60/4/43.2	60/3.2/31.7
Skull	Lat	L	70/2/28.0	63/2.8/41.2	70/8/167.6	60/4/63.6	60/3.2/42.1
Chest	AP		66/1.8/32.0	81/1.25/47.1	70/2.5/81.7	60/1.2/15.5	70/1.6/29.3
Chest	Obl.Ribs	R	73/2/50.3	81/1.25/49.7	70/5/123.1	60/1.2/17.6	65/1.6/15.8
Chest	Obl.Ribs	L	73/2/50.2	81/1.25/50.0	70/5/118.6	60/1.2/18.5	65/1.6/17.5
Chest	Lat	L	-	81/2.5/91.2	75/3.2/109.5	-	70/1.6/24.5
Sternum	Lat	L	-	-	60/5/6.9	-	65/2.5/15.4
Abdomen/pelvis	AP		63/4/62.8	64.5/2/65.6	70/3.2/120.9	65/1.6/31.8	65/2.5/51.7
Spine	Lat C-spine	L	64.5/5/35.2	-	60/2/17.9	-	-
Spine	Lat T-spine	L	70/2.8/19.3	-	60/5/68.0	-	65/4/30.0
Spine	Lat L-spine	L	68/3.2/20.23	60/2.5/21.5	60/5/46.2	-	65/4/30.1
Spine	Lat T- & L-spine	L	70/3.2/19.7	-	60/5/80.6	65/5/75.7	65/4/57.6
Humerus	AP	R	55/1.6/8.5	50/1/7.8	65/2/25.1	55/1.1/6.3	55/2/9.4
Forearm	AP	R	55/1.4/5.9	50/1/1.8	65/1.6/16.2	55/1.1/4.0	55/1.25/5.3
Hand	DP	R	50/1.25/6.3	50/1/4.8	60/1.25/11.4	55/1.1/5.3	55/1/5.3
Coned elbow	Lat	R	55/1.4/3.4	50/1/3.3	60/2/6.2	55/1.1/3.0	55/2/8.1
Coned wrist	Lat	R	50/1.3/1.2	50/1/2.7	60/1.6/4.4	55/1.1/3.3	55/2/6.2
Humerus	AP	L	55/1.6/6.1	50/1/8.0	65/2/25.3	55/1.1/6.8	55/2/11.8
Forearm	AP	L	55/1.4/4.3	50/1/3.0	65/1.6/16.7	55/1.1/4.5	55/1.25/6.2
Hand	DP	L	50/1.25/3.0	50/1/4.2	60/1.25/11.6	55/1.1/5.0	55/1/3.8
Coned elbow	Lat	L	55/1.5/2.9	50/1/3.2	60/2/5.9	55/1.1/3.4	55/2/7.4
Coned wrist	Lat	L	50/1.3/1.4	50/1/2.3	60/1.6/4.4	55/1.1/3.2	55/2/5.5
Femur	AP	R	58.5/2/24.9	53.5/1.45/16.1	65/3.2/66.5	57/1.2/12.0	65/2.5/36.7
Tibia & fibula	AP	R	55/1.6/10.7	53.5/1.3/11.1	65/2/25.6	57/1.2/10.0	55/2/12.4
Foot	DP	R	50/1.25/3.9	52/1.3/4.5	60/1.6/10.0	57/1.2/5.2	52/1/4.3
Coned knee	AP	R	55/1.7/6.1	52/1.45/7.3	65/2.5/16.5	57/1.2/5.6	55/2/11.8
Coned ankle	AP	R	52/1.7/4.1	52/1.3/3.7	60/1.6/6.8	57/1.2/4.3	55/1.6/5.7
Coned knee	Lat	R	55/1.7/7.1	52/1.4/8.5	65/2.5/22.6	57/1.2/6.2	60/2.5/12.2
Coned ankle	Lat	R	52/1.7/3.4	52/1.3/4.6	60/1.6/9.5	57/1.2/5.1	55/1.6/6.8
Femur	AP	L	58.5/2/24.8	53.5/1.45/17.5	65/3.2/66.9	57/1.2/12.2	65/2.5/36.6
Tibia & fibula	AP	L	55/1.6/11.1	53.5/1.3/13.1	65/2/25.7	57/1.2/10.0	55/2/12.4
Foot	DP	L	50/1.25/3.3	52/1.3/4.6	60/1.6/8.4	57/1.2/5.1	52/1/4.3
Coned knee	AP	L	55/1.7/7.9	52/1.45/5.2	65/2.5/16.6	57/1.2/5.8	55/2/12.2
Coned ankle	AP	L	52/1.7/4.5	52/1.3/3.6	60/1.6/6.6	57/1.2/4.5	55/1.25/4.5
Coned knee	Lat	L	55/1.7/6.7	52/1.4/4.4	65/2.5/3.0	57/1.2/7.3	55/2/11.8
Coned ankle	Lat	L	52/1.7/4.9	52/1.3/7.4	60/1.6/11.4	57/1.2/5.0	55/1.6/4.6
Femur	AP	Both	-	-	65/3.2/141.5	-	65/2.5/91.8
Tibia & fibula	AP	Both	-	-	65/2/1.0	-	55/3.2/36.6

*AP* anteroposterior, *C-spine* cervical spine, *DP* dorsoplantar, *kVp* peak kilovoltage, *L* left, *Lat* lateral, *L-spine* lumbar spine, *mAs* milliampere per second, *Obl.Ribs* oblique ribs, *PA* posteroanterior, *R* right*,* *SID* source to image distance, *T-spine* thoracic spine

### Calculation of effective dose

A summary of the cumulative KAP values and effective doses calculated using the air kerma multiplied by the scaled-down irradiated area for a 2-year-old child for a radiographic skeletal survey for each department is presented in Table 5. As it is assumed that all images are diagnostic as a minimum, then the mean would be artificially skewed by the higher doses because there is a natural clipping at the low end (in other words, these would not be normally distributed if 100 clinical centres had been sampled, rather than 5). Therefore, the median value has been presented.

**Table 5:** Cumulative air kerma area product (KAP) and effective doses for a 2 year old estimated from the phantom studies performed in five radiology departments

	RCR protocol		Local SPA protocol	
Radiology site	Estimated cumulative KAP (mGy·cm^2^)	Effective dose (mSv)	Estimated cumulative KAP (mGy·cm^2^)	Effective dose (mSv)
1	246	0.09	-	-
2	227	0.09	221	0.11
3	667	0.33	592	0.35
4	220	0.05	-	-
5	238	0.08	199	0.07
Median	238	0.09	221	0.11

*RCR* Royal College of Radiologists, *mSv* millisievert, *SPA* suspected physical abuse

### Calculation of radiation-induced cancer and death risks

A summary of the estimated risk of radiation-induced cancer and death calculated using the BEIR VII model for a radiographic skeletal survey according to the RCR protocol for each department is presented in Table 6.

**Table 6:** Estimated radiation-induced cancer risks and estimated mortality risks for a 2 year old using the Biologic Effects of Ionizing Radiation (BEIR) VII model

	BEIR VII (risk of radiation-induced cancer)		BEIR VII (risk of radiation-induced death)	
	2 year old boy	2 year old girl	2 year old boy	2 year old girl
Radiology site	1 in…			
1	50,000	30,000	110,000	70,000
2	50,000	30,000	110,000	70,000
3	10,000	10,000	30,000	20,000
4	90,000	50,000	200,000	120,000
5	60,000	30,000	120,000	80,000
Median	50,000	30,000	110,000	70,000
Median for a 2 year old	40,000		90,000	

## Discussion

Whilst radiation dose and image quality are inextricably linked, this study focused on establishing the typical total effective doses for a radiographic skeletal survey across a range of sites where equipment and local protocols differ. Due to the range of 16–31 projections acquired in each department, establishing an overarching measurement metric is challenging. This was minimized by also performing the RCR protocol for initial skeletal surveys, so radiation dose could be compared between sites. As the preprogrammed radiographic exposure factors used at each site are those used to image children who present for clinically indicated and justified radiographic examinations, it is assumed that the resultant image quality is diagnostic according to the local radiologists. Whilst the irradiated fields for the projections differ between individual radiographers, the irradiated field is still collimated to the anatomical regions of interest in accordance with local protocols, so this is reflective of clinical practice.

Table 3 shows that each of the three children’s departments (sites 2, 3 and 5) performed fewer projections than those recommended by the RCR. Sites 3 and 5 exposed both of the legs at the same time, i.e. one projection including both right and left femurs. Whilst this was justified as an effort to optimise the radiographic examination and reduce radiation exposure to the child, coned projections of the joints where the perpendicular X-ray beam is centred over the joint are not performed routinely. Thus, subtle fractures including bucket handle or corner metaphyseal injuries may be missed due to the diverging X-ray beam projecting the metaphyses onto the image receptor [30]. Radiographers performing the SPA imaging must have a good understanding of the clinical challenges associated with imaging children to ensure that pathology is not missed. This is why the images should ideally be reviewed by a radiologist before the child leaves the department in case further imaging is required; the RCR recommends within 24 h [2]. Ideally, paediatric training including knowledge of the local medicolegal system should be undertaken by radiographers involved in imaging SPA cases [31].

Published literature suggests that the radiation doses associated with skeletal survey radiographs are varied but appear to be reducing over time, which would be consistent with improvements in technology. Effective doses for skeletal surveys have decreased over the last decade from being greater than 1 mSv in 2008 to being approximately 0.2 mSv today [8, 32, 33]. This is consistent with the more widespread installation of digital radiography (DR) units. All sites included in this phantom study have DR equipment and when comparing the radiation doses if following the RCR protocol, the median effective dose measuring 0.09 mSv (Table 5) is less than half (45%) of the effective dose calculated by Rao et al. [8] and approximately 80% of the radiation dose of 0.11 mSv associated with a return flight from Melbourne to London [34] or approximately one-twentieth (5%) of the annual background radiation dose of 1.7 mSv in Australia [35].

When looking more closely at the individual sites in Table 4, review of the preprogrammed exposure factors at site 4 suggests that they may not have been optimized for individual projections, therefore we assume that radiographers do not use the preprogrammed exposure factors but manually set their own exposures based on their individual clinical experience. It is recommended that the image quality at such a low dose be reviewed to determine that the clinical images obtained using these preprogrammed exposure factors are diagnostic and don’t lead to projections being frequently repeated. The selection of such low exposure factors corresponds with site 4 (Table 5) having the lowest effective dose at 0.05 mSv, which is one-third (33%) of the dose reported by Wang et al. [32] for children younger than 3 years old. Site 3 in Table 5 has the highest effective dose, which at 0.33 mSv is 1.5 times (150%) higher than that published by Rao et al. [8]. Equipment from the same vendor is used at site 5 where the effective dose is 0.08 mSv (i.e. 25% of the dose delivered at site 3). Review of the exposure factors recorded for each projection at each of the sites in Table 4 shows that the kV_p_ and mAs for each projection are noticeably higher at site 3. When compared to site 5, there is often a difference of approximately 5–10 kV_p_ and almost double the mAs for each projection. It was considered that perhaps the selection of these exposure factors may be linked to the use of an anti-scatter grid, but site 3 confirmed that a grid was not used. The other noticeable difference between sites 3, 4 and 5 was the SID used; at site 3, the SID was 100 cm, whereas at site 4 it was 115 cm and 120 cm at site 5. As both sites 3 and 5 use equipment from the same vendor and regularly image children, it is recommended that site 3 undertakes an optimization programme and review of the exposure factors and SID to see if they can be improved without detracting from the image quality required for confident reporting of SPA cases by the radiologists. It is also important for site 4 to review their doses and assess image quality to verify that their exposures are not too low.

A wide range of radiation doses (0.7–0.35 mSv) was delivered when the local departmental protocols were applied (Table 5). This is most likely due to the different combinations of projections used between the RCR protocol and the local SPA protocol. Site 2 is the only dedicated children’s hospital that images each part of the lower limb separately, thereby taking into consideration the effects of the diverging X-ray beam on the detection of subtle metaphyseal fractures. Table 5 also presents the cumulative KAP values for each site, which is more applicable clinically for radiographers, whereas the effective dose estimates are of value when providing information relating to radiation risks as part of the informed consent process.

**Fig. 6:**
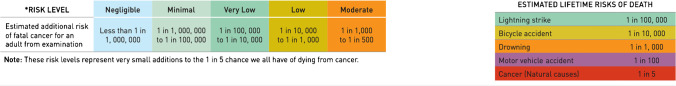
Putting radiation-induced death risks into perspective, noting that these are for adults not children. Reproduced with permission from ARPANSA Fact Sheet – Medical Imaging: Information for Patients [36]. Commonwealth of Australia as represented by the Australian Radiation Protection and Nuclear Safety Agency (ARPANSA)

Table 6 presents the estimated risks of radiation-induced cancer associated with the radiation dose received by 2-year-old children who have a radiographic skeletal survey, with the median risk being higher for girls (1 in 30,000) compared to boys (1 in 50,000). According to Fig. 6, these very low risks are in addition to the 1 in 5 baseline risk of naturally developing cancer in a lifetime [36]. It is also noted that there is “no evidence of human health effects” at radiation doses below 10 mSv [35]. Putting these very low additional radiation risks into perspective for those providing informed consent is a vital component of ensuring families are making an informed decision, especially for screening siblings in SPA cases (if informed consent is withheld, the examination cannot proceed without order by a court of law, who will only act with regard to the best interests of the child). The risks of radiation-induced cancer can be compared to the fact that 1 in 20,000 children younger than 1 year old and 1 in 165,000 ages 1 to 4 years died from SPA in Victoria in 2016 [15].

Table 6 also compares the risk estimates of radiation-induced death or fatal cancer with the median additional risk of dying from radiation-induced cancer across the five sites, being minimal for 2-year-old boys and very low for 2-year-old girls. The 1 in 10,000 risk of dying from a bicycle accident or 1 in 100,000 risk of dying from being struck by lightning (Fig. 6) [36] should be considered when helping a caregiver put the risks into perspective. This should also be considered in relation to the estimated risk of 1 in 1,900 children younger than 5 years old in Australia being diagnosed with a blood cancer or 1 in 1,000 risk of developing any type of cancer [6]. The risk of radiation-induced death can be contextualised with deaths from SPA in 2016 reported to have occurred in 1 in 20,000 children younger than 1 year old and 1 in 165,000 ages 1 to 4 years old [15].

The BEIR VII model estimates are based upon a United States of America population, and it is important to acknowledge the advice of the BEIR VII committee that “the risk estimates should be regarded with a healthy scepticism, placing more emphasis on the magnitude of the risk” [29]. It is clear from Table 6 that the median additional risk of death from radiation-induced cancer for a 2-year-old child is overall very low (1 in 90,000).

Limitations of the research include the fact that the KAP measurements recorded at each site had to be scaled down mathematically to calculate the air kerma values and resultant effective doses for a 2 year old. This was unavoidable as a 5-year-old bone phantom is the only SPA phantom commercially available.

In another study, routine whole-body skeletal survey CT scans of the same 5-year-old anthropomorphic phantom at two sites (each with a different CT scanner), delivered an estimated radiation dose of 2.78 mSv and 0.18 mSv using a 16-cm phantom and 5.66 mSv and 0.37 mSv using a 32-cm phantom [37]. The mean effective dose in a case series published by Lawson et al. [38], in which all children were ages 8 months old or younger, was 1.18 mSv; the authors will now conduct a phantom study aimed at acquiring a diagnostic low-dose CT skeletal survey protocol yielding an effective radiation dose similar to that of the radiographic series.

## Conclusion

Having acquired the projections recommended by the RCR for an initial skeletal survey to investigate suspected physical abuse, the median effective radiation dose to the phantom was 0.09 mSv across the five radiology sites, which results in a very low additional risk of radiation-induced cancer (1 in 40,000). The additional radiation-induced death (1 in 90,000) associated with 0.09 mSv is equivalent to the risk of dying from being stuck by lightning. The authors will use these results to acquire a diagnostic low-dose CT skeletal survey with a similar effective radiation dose of 0.1–0.2 mSv.

## Supplementary Information

Below is the link to the electronic supplementary material.

Supplementary file1 (DOCX 16 KB)

Supplementary file2 (DOCX 29 KB)
