# Supplementary material for: Typical median effective radiation doses using an anthropomorphic bone fracture phantom for initial radiographic skeletal surveys in the investigation of suspected physical abuse
Source: Pediatr Radiol. 2022 Aug 22;53(1):57–68. doi: 10.1007/s00247-022-05456-x (PMC9816253; doi:10.1007/s00247-022-05456-x)
Supplement: Supplementary file 1 — Supplementary file1 (DOCX 16 KB) [file 247_2022_5456_MOESM1_ESM.docx]

**Online Supplementary Material 1** Exposure factors used to calibrate the mobile kerma area product meter (PTW Diamentor) at each of the radiology sites, ranging from 50-81 kV_p_ to 1-4 mAs

| **kV** | **mAs** | **kV** | **mAs** | **kV** | **mAs** |
| --- | --- | --- | --- | --- | --- |
| 50 | 1 | 60 | 1.25 | 70 | 2 |
| 50 | 1.25 | 60 | 1.6 | 70 | 2.5 |
| 50 | 1.32 | 60 | 2 | 70 | 2.8 |
| 52 | 1.3 | 60 | 2.5 | 70 | 3.2 |
| 52 | 1.4 | 60 | 5 | 70 | 5 |
| 52 | 1.45 | 61.5 | 2.5 | 70 | 8 |
| 52 | 1.5 | 63 | 2.8 | 73 | 2 |
| 52 | 1.6 | 63 | 4 | 75 | 3.2 |
| 52 | 1.68 | 64.5 | 2 | 81 | 1.25 |
| 53.5 | 1.3 | 64.5 | 4 |  |  |
| 53.5 | 1.4 | 65 | 1.25 |  |  |
| 53.5 | 1.45 | 65 | 1.6 |  |  |
| 55 | 1 | 65 | 2 |  |  |
| 55 | 1.4 | 65 | 2.5 |  |  |
| 55 | 1.49 | 65 | 3.2 |  |  |
| 55 | 1.6 | 66 | 1.8 |  |  |
| 55 | 1.68 | 66 | 3.2 |  |  |
| 55 | 1.7 |  |  |  |  |
| 58.5 | 2 |  |  |  |  |

*kVp* peak kilovoltage, *mAs* milliampere per second
