# Supplementary material for: Typical median effective radiation doses using an anthropomorphic bone fracture phantom for initial radiographic skeletal surveys in the investigation of suspected physical abuse
Source: Pediatr Radiol. 2022 Aug 22;53(1):57–68. doi: 10.1007/s00247-022-05456-x (PMC9816253; doi:10.1007/s00247-022-05456-x)
Supplement: Supplementary file 2 — Supplementary file2 (DOCX 29 KB) [file 247_2022_5456_MOESM2_ESM.docx]

**Online Supplementary Material 2** Published literature summarising radiation dose for skeletal survey radiographs

| *Authors* | *Country* | *Age (yrs)* | *Projections* | *DRL (mGy)* | *Effective dose*  *(mSv)* |
| --- | --- | --- | --- | --- | --- |
| Royal College of Radiologists and Royal College of Paediatrics and Child Health (2008) [1] | UK |  |  |  | **0.9-1.8** |
| Sonawane et al. (2011) [2] | India | 1-4 | AP/PA CXR  Lat CXR  Lat T-sp  Lat L-sp  AXR  Pelvis  AP SXR  Lat SXR | 0.28  0.42  -  -  0.44  0.40  0.43  0.38  **2.38** |  |
| Matthews et al. (2014) [3] | Ireland | 1-5 | CXR  AXR  Pelvis | 1.53  11.6  4.99  DAP: **18.12** cGy·cm^2^  (181.2 mGy·cm^2^) |  |
| Bainbridge et al. (2015) [4] | UK | 0-2 | Skeletal survey |  | **0.8** |
| American College of Radiology (ACR) and Society for Pediatric Radiology (SPR) (2016) [5] | USA | 0-5 | Skeletal survey |  | **0.3-3** |
| Berger et al.(2016) [6] | USA | 0-1 | Skeletal survey (15 projections, <1 year old) |  | **0.2** |
| Paulo et al. (2015) [7] | Portugal | 1-5 | CXR  AXR  Pelvis | 17  84  67  KAP: 168 mGy·cm^2^ |  |
| Nguyen & Hart 2018 [8] | (Australia)  Literature review | 0-1  0-2 | Skeletal survey  Skeletal survey |  | 0.2  **0.8** |
| Rao R et al. (2019) [9] | New Zealand | 0  1  2 | Skeletal survey (17-22 projections) |  | 0.17  0.23  0.28  **0.2** |
| Celier et al. (2020) [10] | France | 1-5  3 months-5 years  4-8 years | CXR  AXR  Pelvis | 19  79  120  KAP: 218 mGy·cm^2^ |  |
| Mussmann et al. (2020) [11] | Denmark | 1-2 years | Skeletal survey (33 projections) | DAP: 779 mGy·cm^2^ | **0.18** |

*AP* anteroposterior, *AXR* abdominal radiograph, *CXR* chest radiograph, *DAP* dose area product, *DRL* diagnostic reference level, *KAP* kerma area product, *L-sp* lumbar spine radiograph, *Lat* lateral, *mGy* milli Gray, *mSv* millisievert, *mths* months, *PA* posteroanterior; *SXR* skull radiograph, *UK* United Kingdom, *USA* United States of America, *T-sp* thoracic spine radiograph

**References**

1. The Royal College of Radiologists, The Royal College of Paediatrics and Child Health (2008) Standards for radiological investigations of suspected non-accidental injury. <http://www.rcr.ac.uk/docs/radiology/pdf/RCPCH_RCR_final.pdf> Accessed 29 June 2022

2. Sonawane AU, Sunil Kumar JVK, Singh M, Pradhan AS (2011) Suggested diagnostic reference levels for paediatric X-ray examinations in India. Radiat Prot Dosimetry 147: 423-428

3. Matthews K, Brennan PC, McEntee MF (2014) An evaluation of paediatric projection radiography in Ireland. Radiography 20:89-194

4. Bainbridge JK, Huey BM, Harrison SK (2015) Should bone scintigraphy be used as a routine adjunct to skeletal survey in the imaging of non-accidental injury? A 10 year review of reports in a single centre. Clin Radiol 70:e83-e89

5. American College of Radiology (ACR), Society for Pediatric Radiology (SPR) (2016) ACR–SPR Practice parameter for the performance and interpretation of skeletal surveys in children. <https://www.acr.org/-/media/ACR/Files/Practice-Parameters/Skeletal-Survey.pdf> Accessed 29 June 2022

6. Berger RP, Panigrahy A, Gottschalk S, Sheetz M (2016) Effective radiation dose in a skeletal survey performed for suspected child abuse. J Pediatr 171:310-312

7. Paulo G, Vaño E, Rodrigues A (2015) Diagnostic reference levels in plain radiography for paediatric imaging: A Portuguese study. Radiography 22:e34-e39

8. Nguyen A, Hart R (2018) Imaging of non‐accidental injury -what is clinical best practice? J Med Radiat Sci 65:123-130

9. Rao R, Browne D, Lunt B et al (2019) Radiation doses in diagnostic imaging for suspected physical abuse. Arch Dis Child 104:863-868

10. Célier D, Roch P, Etard C et al (2020) Multicentre survey on patient dose in paediatric imaging and proposal for updated diagnostic reference levels for France. Part 2: plain radiography and diagnostic fluoroscopy. Eur Radiol 30:1182-1190

11. Mussmann B, Hardy M, Rajalingam R et al (2021) Local diagnostic reference levels for skeletal surveys in suspected physical child abuse. Radiography 27:425-429
